# Whole‐genome variant analysis of Alzheimer's disease biomarker traits

**DOI:** 10.1002/alz70855_099640

**Published:** 2025-12-23

**Authors:** Xuewen Xiao, Xinxin Liao, Yiliang Liu, Shilin Luo, Beisha Tang, Jinchen Li, Bin Jiao, Lu Shen

**Affiliations:** ^1^ Xiangya Hospital, Central South University, Changsha, Hunan, China

## Abstract

**Background:**

Genetics plays a significant role in Alzheimer's disease (AD) biomarker traits. However, the underlying genetic underpinnings remain poorly understood.

**Methods:**

A total of 3,707 individuals were enrolled, including 1,697 AD patients and 2,010 controls, all of whom underwent whole genome sequencing. Cerebrospinal fluid (CSF) biomarkers were available in 226 AD patients, while plasma biomarkers were measured in 302 samples (184 AD patients and 118 controls). We performed association analysis for common variants (MAF ≥ 0.05) and gene‐based association studies for rare variants (MAF < 0.05) with AD biomarker traits. Additionally, we constructed a polygenic risk score (PRS) to evaluate its association with AD risk and biomarkers.

**Result:**

PRS was significantly associated with higher AD risk, lower MMSE scores, and elevated levels of plasma *p*‐tau217 and GFAP. Gene‐based rare variant association studies revealed that three novel genes reached genome‐wide significance, including *ABCA7* rare damaging variants for MMSE score, an *ECSIT* rare damaging missense variant for CSF Aβ42, and an *ADPRHL1* rare LoF variant for plasma GFAP, respectively (all *p* <0.05).

**Conclusions:**

Our study highlights the contribution of both PRS and rare variants in *ABCA7, ECSIT*, and *ADPRHL1* to AD biomarker traits. These findings provide insights into the pathophysiology of AD and may lead to novel targets for diagnosis and therapy.

